# Prevalence, Phenotypes, and Comorbidities of Polycystic Ovary Syndrome Among Indian Women

**DOI:** 10.1001/jamanetworkopen.2024.40583

**Published:** 2024-10-23

**Authors:** Mohd Ashraf Ganie, Subhankar Chowdhury, Neena Malhotra, Rakesh Sahay, Prasanta Kumar Bhattacharya, Sarita Agrawal, P. K. Jabbar, Vanita Suri, Roya Rozati, Vishnubhatla Sreenivas, Mohammad Salem Baba, Imtiyaz Ahmad Wani, Haroon Rashid, Abhilash Nair, Amlin Shukla, Taruna Arora, Bharati Kulkarni

**Affiliations:** 1 Department of Endocrinology, Sher-i-Kashmir Institute of Medical Sciences, Srinagar, India; 2 Department of Clinical Research, Sher-i-Kashmir Institute of Medical Sciences, Srinagar, India; 3 Department of Endocrinology Metabolism, Institute of Postgraduate Medical Education Research, Kolkata, India; 4 Department of Obstetrics and Gynaecology, All India Institute of Medical Sciences, New Delhi, India; 5 Department of Endocrinology, Osmania Medical College, Hyderabad, India; 6 Department of General Medicine, North-Eastern Indira Gandhi Regional Institute of Health and Medical Sciences, Shillong, India; 7 Department of Obstetrics and Gynaecology, All India Institute of Medical Sciences, Raipur, India; 8 Department of Endocrinology, Government Medical College, Thiruvananthapuram, India; 9 Department of Obstetrics and Gynaecology, Postgraduate Institute of Medical Education and Research, Chandigarh, India; 10 Department of Obstetrics and Gynaecology, Maternal Health, Research Trust, Hyderabad, India; 11 Department of Biostatistics, All India Institute of Medical Sciences, New Delhi, India; 12 Reproductive Biology and Maternal Health, Child Health, Indian Council of Medical Research, New Delhi, India

## Abstract

**Importance:**

The prevalence of polycystic ovary syndrome (PCOS) varies across the globe. Indian studies on PCOS are limited by poor design, small sizes, regional representations, and varying methods.

**Objectives:**

To estimate the nationwide prevalence of PCOS in India, examine the phenotypic spectrum, and assess the magnitude of comorbidities associated with PCOS.

**Design, Setting, and Participants:**

This cross-sectional study recruited 9824 women aged 18 to 40 years from November 1, 2018, to July 31, 2022, across 5 zones of the country. A prevalidated questionnaire dichotomized women into screen-positive and screen-negative groups. Relevant clinical, hormonal, and sonographic assessments categorized women as either women with criteria-based PCOS (ie, National Institutes of Health [NIH] 1990 criteria, Rotterdam 2003 criteria, or Androgen Excess and Polycystic Ovary Syndrome Society [AE-PCOS] criteria), women with partial phenotypes (hyperandrogenism, oligomenorrhea, or polycystic morphology labeled as pre-PCOS), or healthy women, in addition to quantitating various comorbidities.

**Main Outcomes and Measures:**

The prevalence and phenotypes of PCOS among women of reproductive age and the burden of comorbidities associated with PCOS.

**Results:**

A total of 8993 women (mean [SD] age, 29.5 [6.2] years) were enrolled in this study; 196 women were already diagnosed with PCOS, 2251 were categorized as screen positive, and 6546 were categorized as screen negative. The mean (SD) age of screen-positive women (28.1 [6.4] years) was lower than that of screen-negative women (29.7 [6.1] years) (*P* < .001), and the mean (SD) age at menarche was higher in the former group (13.2 [1.3] vs 13.1 [1.2] years; *P* < .001). The national prevalence of PCOS was 7.2% (95% CI, 4.8%-10.8%) by NIH 1990 criteria, 19.6% (95% CI, 12.7%-29.2%) by Rotterdam 2003 criteria, and 13.6% (95% CI, 8.4%-21.6%) by AE-PCOS criteria. Overall, PCOS phenotypes C (501 [40.8%]) and D (301 [24.6%]) were the most common, and 492 women (pre-PCOS subgroup) had oligomenorrhea (n = 75), hyperandrogenism (n = 257), or polycystic ovarian morphology (n = 160) only. Among women with PCOS (n = 1224), obesity was present in 529 (43.2%), dyslipidemia in 1126 (91.9%), nonalcoholic fatty liver disease in 403 (32.9%), metabolic syndrome in 305 (24.9%), impaired glucose tolerance in 111 (9.1%), diabetes in 41 (3.3%), and hypertension in 101 (8.3%). The pre-PCOS subgroup (n = 492) displayed similar metabolic aberrations (dyslipidemia: 390 [79.3%]; metabolic syndrome: 78 [15.9%]; nonalcoholic fatty liver disease: 163 [33.1%]; impaired glucose tolerance: 62 [12.6%]; diabetes: 7 [1.4%]; and hypertension: 26 [5.3%]).

**Conclusions and Relevance:**

In this cross-sectional study of reproductive-age women recruited across India, the prevalence of PCOS was high, with phenotype C being predominant. Most of these women had metabolic abnormalities. These findings are crucial for developing preventive and therapeutic strategies, potentially integrating PCOS management into national health programs.

## Key Points

**Question:**

What is the prevalence, variation of phenotypes, and comorbidities associated with polycystic ovary syndrome (PCOS) in India?

**Findings:**

In this cross-sectional study of 9824 women aged 18 to 40 years, the weighted national prevalences of PCOS by the National Institutes of Health 1990 and Rotterdam 2003 criteria were 7.2% and 19.6%, respectively, and PCOS phenotype C was the most prevalent (40.8%). Among women with PCOS, 43.2% had obesity, 91.9% had dyslipidemia, 32.9% had nonalcoholic fatty liver disease, 24.9% had metabolic syndrome, 3.4% had diabetes, and 8.3% had hypertension.

**Meaning:**

These findings suggest that there is a high prevalence of PCOS among women in India, with the majority of these women having 1 or more metabolic diseases.

## Teaser

This cross-sectional study of women with polycystic ovary syndrome in India assesses the prevalence, phenotypes, and associated comorbidities of the syndrome.

## Introduction

Polycystic ovary syndrome (PCOS) is characterized by menstrual irregularities, hyperandrogenism, and polycystic ovarian morphology (PCOM).^1,2^ Beyond its implications for reproductive health, PCOS is linked to an array of metabolic comorbidities, such as obesity, metabolic syndrome, insulin resistance, dysglycemia, and nonalcoholic fatty liver disease (NAFLD),^3,4,5,6,7,8,9,10,11,12,13^ mandating a variety of treatment options targeting symptoms or dominant pathologic mechanisms.^14,15,16^ Consequent to diverse clinical features, different criteria are used for diagnosing PCOS.^17,18,19^ Globally, the prevalence of PCOS ranges from 4% to 21%,^20,21,22,23,24,25,26,27,28,29^ and in India, the prevalence varies from 2% to 35%.^30,31,32,33,34,35,36^ Factors such as population ethnicity, geographic location, diagnostic criteria, and differences in androgen and ultrasonography-based assessments contribute to the variability in PCOS prevalence.^37^ However, there is still a lack of well-designed, population-based studies with sufficient sample sizes to accurately determine the true prevalence of PCOS. Given the substantial impact of PCOS on fertility, noncommunicable diseases, and potential transgenerational effects, this study aimed to assess the prevalence of PCOS across India, examine its phenotypes, and estimate associated comorbidities.

## Methods

This nationwide, multicenter, epidemiologic, cross-sectional study covered 5 zones of India (North, Northeast, East, Central, and South) and included participants from 8 states. Recruitment occurred between November 1, 2018, and July 31, 2022. The uniform, comprehensive study protocol, published elsewhere,^38^ used a predesigned, pilot-tested screening questionnaire on community-dwelling women, aged 18 to 40 years, selected via a stratified sampling procedure using voter identification cards of the 2011 Census of India. Individuals who had oligomenorrhea, secondary amenorrhea, and clinical hyperandrogenism were defined as screen positive (probable PCOS), whereas those without any such feature were defined as screen negative (healthy). Women previously diagnosed with PCOS were confirmed by verifying their medical records. The study was conducted in accordance with the Declaration of Helsinki,^39^ and the study protocol was reviewed and approved from all the participating institutional ethics committees. Written informed consent was obtained from all the participants before their enrollment. The study followed the Strengthening the Reporting of Observational Studies in Epidemiology (STROBE) reporting guideline.

### Study Design

From each participating site, 1 rural and 1 urban Vidhan Sabha (assembly) constituency was selected. Subsequently, 8 to 10 polling booths were randomly chosen from each. Women aged 18 to 40 years listed in the voter identification list (obtained from respective electoral offices as per the 2011 Census of India) were approached. Eligible participants were those who had lived in the area for at least 1 year, were neither lactating nor pregnant, and were willing to participate. Trained research staff conducted face-to-face interviews, capturing information using a validated questionnaire. Data collected included age, marital status, menstrual cyclicity (age of menarche and cycle interval, length, and flow), parity (if married), hirsutism, acne, alopecia, and relevant history. Women with chronic cardiovascular, liver, lung, neurologic, kidney, and musculoskeletal disorders or taking medications such as insulin sensitizers, steroids, antiandrogens, oral contraceptives, antidepressants, lipid-lowering agents, or other drugs affecting glucose tolerance, insulin sensitivity, and androgen metabolism, were excluded (exclusion criteria did not apply to women already diagnosed with PCOS). After exclusions, participants underwent transabdominal ultrasonography and hormonal analysis. Polycystic ovarian syndrome was diagnosed by the National Institutes of Health (NIH) 1990 criteria,^17^ Rotterdam 2003 criteria,^18^ or Androgen Excess and Polycystic Ovary Syndrome Society 2009 (AE-PCOS) criteria.^19^

### Clinical Examination and Imaging

All the questionnaire-positive women and 20% of the questionnaire-negative women were invited to the respective institutes for full evaluation on a specified day. Evaluation of hirsutism, androgenic alopecia, and acne vulgaris was performed by a single trained observer at each site, with inter se agreement tested at each respective site. Hirsutism was scored by the modified Ferriman-Gallwey method.^40^ Acneiform lesions (comedones, papules, pustules, nodules, abscesses, cysts, and scars) were graded per the Leeds technique (ie, 0 indicating no acne; 1, comedones and few papules on the face; 2, comedones, papules, and a few pustules on the face; and 3, larger inflammatory papules, pustules, and a few cysts involving the face, neck, and upper portions of the trunk).^41^ Androgenic alopecia was graded from 1 to 3 as per the Ludwig scale.^42^ Transabdominal ultrasonography (GE-LOGIQ P5 PRO, General Electric Company) was performed to demonstrate any evidence of PCOM. The inter se agreement between the clinicians and the sonologists was obtained before the initiation of the study and was within acceptable limits.

### Laboratory Evaluation

Blood samples from eligible women were collected in the morning (8:00 to 9:00 am) after an overnight (8-12 hours) fast on days 2 to 7 of the spontaneous or medroxyprogesterone-induced menstrual cycle for serum total tetraiodothyronine (TT_4_), thyrotropin, luteinizing hormone (LH), follicle-stimulating hormone (FSH), prolactin, total testosterone, estradiol (E_2_), cortisol, 17-hydroxyprogesterone (17-OHP), and dehydroepiandrosterone sulfate (DHEA-S). An oral glucose tolerance test was performed by administering an oral load of 75 g of anhydrous glucose dissolved in 200 to 300 mL of water, and venous blood samples were obtained at baseline (0 minutes) and 60 and 120 minutes after load. Aliquots for hemogram, liver function test results, kidney function test results, and lipid profile were collected from the basal sample. Adrenocorticotrophic hormone–stimulated 17-OHP or overnight dexamethasone-suppressed cortisol test was performed to rule out nonclassic adrenal hyperplasia or Cushing syndrome after obtaining baseline values, wherever necessary.

### Assays

Plasma glucose, serum lipids, uric acid, calcium, phosphorous, liver function tests, and serum kidney function tests were estimated on fully automated biochemistry analyzers at the respective institutes (Beckman Coulter Diagnostics or Siemens). The serum samples for the estimation of hormones such as serum TT_4_, thyrotropin, cortisol, prolactin, LH, FSH, total testosterone, DHEA-S, E_2_, and 17-OHP were transported in a cold chain to the central hormone assay laboratory at the coordinating center. These hormones were measured using the electrochemiluminescence immunoassay on an analyzer (COBAS e-411, Roche Diagnostics) except for 17-OHP, which was assayed using enzyme-linked immunoassay (Diaclone SAS). Sensitivity, specificity, interassay, and intra-assay coefficients of variation were within the prescribed limits as per the respective manufacturer’s protocols.

### Definitions

Oligomenorrhea was defined as fewer than eight menstrual cycles per year or intermenstrual interval greater than 35 days.^1^ Secondary amenorrhea was defined as a cycle length greater than 90 days. Clinical hyperandrogenism was defined as a modified Ferriman-Gallwey score greater than 8 and/or grade 3 acne and/or grade 2 or 3 androgenic alopecia. Biochemical hyperandrogenism was defined as serum total testosterone levels of 0.88 ng/mL or higher (to convert to nanomoles per liter, multiply by 0.0347) and/or serum DHEA-S levels of 246 ng/mL or higher (to convert to micromoles per liter, multiply by 0.027).^43^ The definition of PCOM was the presence of more than 12 peripheral follicles, each measuring 2 to 8 mm in size with echogenic ovarian stroma and/or increased ovarian volume (>10 cm^3^) on transabdominal ultrasonography.^43^

Women who qualified for any of the 3 diagnostic criteria (NIH 1990,^17^ Rotterdam 2003,^18^ or AE-PCOS 2009^19^) were considered to have PCOS after ruling out the relevant disorders. The NIH criteria include the presence of both clinical and/or biochemical hyperandrogenism and oligomenorrhea, whereas the Rotterdam 2003 criteria are based on the presence of 2 of the following: clinical or biochemical hyperandrogenism, oligomenorrhea, and/or PCOM. A case defined by the AE-PCOS criteria should have clinical or biochemical hyperandrogenism and oligomenorrhea or PCOM.^19^ Nonclassic congenital adrenal hyperplasia, Cushing syndrome, androgen-secreting tumors, hyperprolactinemia, hypothalamic amenorrhea, thyroid dysfunctions, and premature ovarian insufficiency were excluded as per the PCOS diagnostic criteria requirements.

On the basis of the Asian criteria, a body mass index of 23 or higher (calculated as weight in kilograms divided by height in meters squared) was considered overweight and 27.5 or higher as obesity, whereas a body mass index of 25 or higher was considered overweight and 30 or higher as obesity using World Health Organization criteria.^44^ Hypertension was considered a systolic blood pressure of 140 mm Hg or higher and/or a diastolic blood pressure of 90 mm Hg or higher.

Dyslipidemia was defined as total cholesterol levels of 200 mg/dL or greater, low-density lipoprotein cholesterol levels of 130 mg/dL or higher, triglycerides levels of 150 mg/dL or higher, and high-density lipoprotein cholesterol levels less than 50 mg/dL (to convert all cholesterol to millimoles per liter, multiply by 0.0259; to convert triglycerides to millimoles per liter, multiply by 0.0113).^45^ Metabolic syndrome was defined as 3 or more of the following; waist circumference greater than 88 cm, blood pressure greater than 130/85 mm Hg, triglycerides level greater than 150 mg/dL, high-density lipoprotein cholesterol level less than 50 mg/dL, or blood glucose fasting greater than 100 mg/dL.^46^ Diabetes was defined as a fasting plasma glucose level of 126 mg/dL or greater or 2-hour postglucose load greater than 200 mg/dL (to convert glucose to millimoles per liter, multiply by 0.0555). Impaired fasting plasma glucose was defined as a fasting plasma glucose level between 100 and 125 mg/dL. Impaired glucose tolerance was defined as a 2-hour post–oral glucose tolerance test glucose value between 140 and 199 mg/dL.^47^

Polycystic ovarian syndrome phenotype A includes hyperandrogenism, oligomenorrhea, and PCOM; phenotype B includes hyperandrogenism and oligomenorrhea; phenotype C includes hyperandrogenism and PCOM; and phenotype D includes oligomenorrhea and PCOM.^48^ Women with only hyperandrogenism, oligomenorrhea, or PCOM with the exclusion of related disorders were considered to have pre-PCOS.^2^

Hyperprolactinemia was defined as a serum prolactin level greater than 52.9 ng/mL (to convert to micrograms per liter, multiply by 1). Overt primary hypothyroidism was defined as a serum thyrotropin level greater than 10 mIU/mL. Overt primary hyperthyroidism was defined as a serum TT_4_ level greater than 14 μg/dL and a thyrotropin level less than 0.1 mIU/mL. Central hypothyroidism was defined as a serum TT_4_ level less than 4 μg/dL and a thyrotropin level less than 10 mIU/mL. Hypothalamic amenorrhea was defined as the absence of menstrual cycles for 90 days, a serum FSH level less than 0.5 mIU/mL, a serum LH level less than 0.5 mIU/mL, and absence of hyperandrogenism. Endogenous Cushing syndrome was defined as a serum cortisol level greater than 30 μg/dL (to convert to nanomoles per liter, multiply by 27.588). Exogenous Cushing syndrome was defined as a serum morning cortisol level less than 1.0 μg/dL. Nonclassic congenital adrenal hyperplasia was defined as a serum 17-OHP level greater than 10 ng/mL. Androgen-secreting tumor was defined as a serum total testosterone level greater than 3 ng/mL (to convert to nanomoles per liter, multiply by 0.0347). Premature ovarian insufficiency was defined as a serum FSH level greater than 40 mIU/mL.^49,50^

### Statistical Analysis

Statistical analysis was performed using Stata software, version 17 (Stata Corp LLC). With a 10% prevalence of PCOS reported in a previous study,^51^ related precision of 1% with a 95% level of confidence, and an assumed attrition rate of 20%, the sample size required was approximately 4500 individuals. Furthermore, given a cluster design and taking a design effect of 2, approximately 9000 individuals were finally enrolled. Prevalence of PCOS among screen-positive and screen-negative groups was calculated after excluding the hormonal comorbidities. The prevalence among criteria-positive participants (NIH, Rotterdam, and AE-PCOS criteria) was determined, and the numbers were projected where information was missing in some variables. Moreover, known cases were added to the total numerator as well as the denominator for calculating the overall prevalence. Prevalence was calculated by site as per area of residence and then combined to get the total, urban, and rural prevalence of PCOS. Prevalence was estimated along with 95% CIs, and cluster adjustments were applied to account for the sampling. Quantitative variables were checked for normality using the Kolmogorov-Smirnoff test. Mann-Whitney U and Kruskal-Wallis tests were used for continuous variables with 2 or more groups in which data were not normally distributed, whereas an unpaired *t* test was used for the ones normally distributed. Categorical variables were compared using the Pearson χ^2^ test. A 2-sided *P* < .05 was considered statistically significant.

## Results

**Figure 1.**
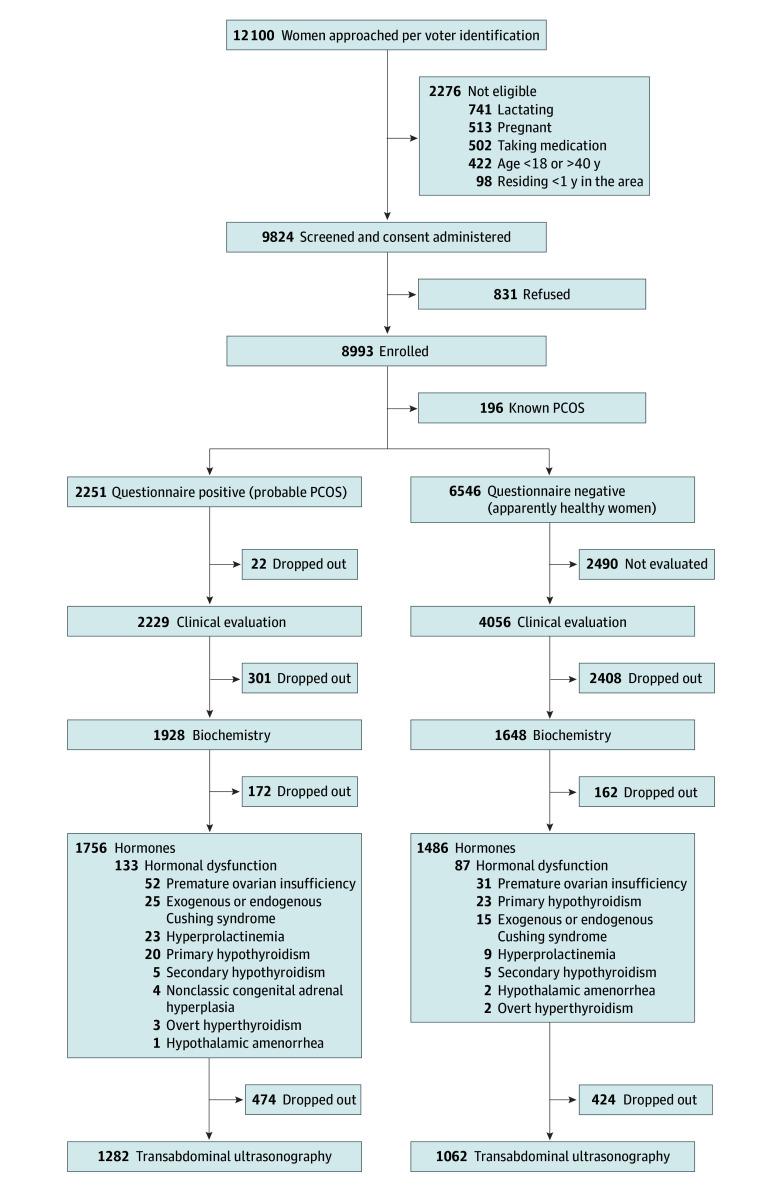
Flow Diagram of the Women Recruited Into the Study PCOS indicates polycystic ovary syndrome.

Of the 12 100 individuals listed in the voter identification list and approached at all the participating sites, 831 refused to participate and 2276 were ineligible. The screening questionnaire was administered to a total of 8993 women (mean [SD] age, 29.5 [6.2] years), of whom 196 had fair documentation of a diagnosis of PCOS and 2251 were categorized as having positive screen results (probable PCOS), leaving 6546 as having negative screen results (healthy). Among probable PCOS, complete hormonal evaluation was available in 1759 women, of whom 133 were excluded because of hormonal dysfunctions. Similarly, among screen-negative women, complete hormonal evaluation was available in 1486 women, of whom 87 were excluded because of hormonal dysfunctions (Figure 1). Although the mean (SD) age of the screen-positive women (28.1 [6.4] years) was lower than that of the screen-negative women (29.7 [6.1] years) (*P* < .001), the age at menarche was higher in the former (13.3 [1.3] vs 13.1 [1.2] years; *P* < .001). The baseline anthropometric, biochemical, and hormonal characteristics are described in Table 1.

**Table 1.** Comparison of Anthropometric, Biochemical, and Hormonal Characteristics of Women With PCOS, Women With Pre-PCOS, and Healthy Women

Characteristic	Mean (SD)		
	Classic PCOS (n = 325)	Pre-PCOS (n = 492)	Healthy (n = 3339)
Age, y	27.4 (6.3)	28.6 (6.4)	29.7 (6.1)
Age at menarche, y	13.2 (1.3)	13.2 (1.3)	13.1 (1.2)
mFGS	9.4 (4.5)	7.6 (4.1)^a^	4.2 (1.5)^b^^,^^c^
Weight, kg	59.4 (11.2)	59.9 (11.1)	58.5 (10.4)
Height, cm	154.8 (6.0)	155.3 (5.6)	156.1 (5.5)
BMI	24.8 (4.5)	24.9 (4.3)	24.7 (4.1)
Bilirubin, mg/dL	0.6 (0.3)	0.6 (0.3)	0.6 (0.3)^b^
Total protein, g/dL	7.4 (0.6)	7.5 (0.6)	7.5 (0.5)
Albumin, mg/dL	4.3 (0.4)	4.4 (0.4)	4.4 (0.4)
AST, U/L	28.3 (13.6)	27.2 (11.8)	25.2 (11.5)^b^
ALT, U/L	26.8 (14.3)	26.0 (14.0)	23.4 (13.4)^b^^,^^c^
ALP, U/L	92.6 (35.8)	91.6 (37.2)	85.6 (31.9)^b^^,^^c^
BGF, mg/dL	89.4 (14.5)	89.9 (15.8)	88.9 (11.4)
Insulin fasting, μIU/mL	18.7 (22.3)	16.0 (17.7)^a^	13.6 (13.6)^b^^,^^c^
Total cholesterol, mg/dL	164.2 (33.3)	164.9 (35.1)	158.4 (28.0)^b^^,^^c^
HDL-C, mg/dL	43.3 (8.4)	44.9 (9.5)	48.7 (10.6)^b^^,^^c^
Triglycerides, mg/dL	124.0 (45.3)	122.9 (44.4)	114.1 (43.6)^b^^,^^c^
LDL-C, mg/dL	96.6 (22.8)	98.5 (25.4)^a^	94.5 (23.8)^c^
Thyrotropin, mIU/L	3.0 (5.1)	3.5 (3.1)	3.0 (3.2)
Prolactin, ng/dL	17.4 (10.6)	16.5 (10.6)	16.1 (9.5)
Cortisol, μg/dL	10.6 (5.3)	10.4 (4.6)	10.6 (6.3)
DHEA-S, μg/dL	182.4 (98.5)	162.0 (87.7)^a^	131.2 (53.2)^b^^,^^c^
LH, mIU/mL	11.3 (9.7)	10.3 (9.2)	10.0 (9.2)
Testosterone, ng/dL	0.4 (0.3)	0.3 (0.2)	0.3 (0.2)^b^
HOMA-IR	73.4 (90.5)	64.4 (73.4)^a^	55.7 (61.6)^b^^,^^c^
FGIR	13.1 (36.4)	12.2 (23.9)	14.6 (28.9)
QUICKI	0.3 (0.1)	0.3 (0.1)	0.3 (0.1)

Abbreviations: ALP, alkaline phosphatase; ALT, alanine transferase; AST, aspartate aminotransferase; BGF, blood glucose fasting; BMI, body mass index (calculated as weight in kilograms divided by height in meters squared); DHEA-S, dehydroepiandrosterone sulfate; FGIR, fasting glucose insulin ratio; HDL-C, high-density lipoprotein cholesterol; HOMA-IR, Homeostatic Model Assessment for Insulin Resistance; LDL-C, low-density lipoprotein cholesterol; LH, luteinizing hormone; mFGS, modified Ferriman-Gallaway score; PCOS, polycystic ovary syndrome; QUICKI, Quantitative Insulin Sensitivity Check Index.

SI conversion factors: To convert bilirubin to micromoles per liter, multiply by 17.104; protein to grams per liter, multiply by 10; albumin to grams per liter, multiply by 10; AST, ALT, and ALP to microkatals per liter, multiply by 0.0167; insulin to picomoles per liter, multiply by 6.945; cholesterol, HDL-C, and LDL-C to millimoles per liter, multiply by 0.0259; triglycerides to millimoles per liter, multiply by 0.0113; prolactin to micrograms per liter, multiply by 1; cortisol to nanomoles per liter, multiply by 27.588; DHEA-S to micromoles per liter, multiply by 0.027; LH to international units per liter, multiply by 1; and testosterone to nanomoles per liter, multiply by 0.0347.

^a^ *P* < .05 for PCOS vs pre-PCOS.

^b^ *P* < .05 for PCOS vs healthy.

^c^ *P* < .05 for pre-PCOS vs healthy.

### Prevalence

The cluster-adjusted prevalence of PCOS was 7.2% (95% CI, 4.8%-10.8%) according to NIH 1990 criteria, 13.6% (95% CI, 8.4%-21.6%) by AE-PCOS criteria, and 19.6% (95% CI, 12.7%-29.2%) by Rotterdam 2003 criteria. Across all criteria, urban areas showed slightly higher prevalence rates (Table 2), with prevalence decreasing with increasing age. When analyzed by zones, Central and North India had the highest prevalences, followed by East and South India, with the lowest prevalence observed in Northeastern India (Table 2).

**Table 2.** Prevalence of Polycystic Ovary Syndrome (PCOS) in India and Its Regional Variation

Zone	Estimated PCOS prevalence, % (95% CI)								
	NIH 1990 criteria			Rotterdam 2003 criteria			AE-PCOS 2009 criteria		
	Urban	Rural	Total	Urban	Rural	Total	Urban	Rural	Total
East	7.8 (5.3-11.2)	8.2 (5.9-11.2)	8.1 (5.6-11.1)	13.6 (10.1-17.5)	16.6 (13.2-20.3)	15.5 (12.4-19.6)	13.1 (9.6-16.9)	14.6 (11.4-18.2)	14.1 (10.9-17.8)
North	8.4 (7.1-9.8)	8.1 (6.8-9.5)	8.2 (6.9-9.6)	27.5 (25.3-29.7)	25.8 (23.8-27.9)	26.4 (24.3-28.5)	15.5 (13.8-17.3)	16.6 (14.8-18.4)	16.2 (14.5-18.0)
South	5.6 (4.4-7.2)	3.0 (2.0-4.3)	3.9 (2.8-5.3)	14.7 (12.7-17.0)	9.2 (7.5-11.3)	11.2 (9.4-13.4)	14.1 (12.1-16.3)	9.0 (7.2-11.0)	10.8 (9.1-13.1)
Central	12.5 (9.8-15.5)	13.1 (10.2-16.4)	12.9 (10.0-16.0)	24.0 (20.4-27.6)	30.7 (26.6-35.0)	28.3 (24.2-32.2)	19.4 (16.1-22.9)	18.8 (15.3-22.5)	19.0 (15.8-22.8)
Northeast	5.7 (3.8-8.2)	2.9 (1.6-4.9)	3.9 (2.4-6.1)	9.4 (7.0-12.4)	6.0 (4.1-8.7)	7.2 (5.1-10.0)	6.2 (4.2-8.6)	3.1 (1.8-5.2)	4.2 (2.6-6.4)
Total	7.8 (7.0-8.7)	6.9 (6.1-7.7)	7.2 (6.4-8.0)	20.3 (19.1-21.5)	19.2 (18.0-20.4)	19.6 (18.3-20.8)	14.3 (13.3-15.5)	13.3 (12.2-14.4)	13.6 (12.6-14.8)
Total cluster adjusted	7.8 (6.0-10.4)	6.9 (4.3-11.0)	7.2 (4.8-10.8)	20.3 (13.5 –29.6)	19.2 (12.2-29.0)	19.6 (12.7-29.2)	14.3 (8.9-22.4)	13.3 (8.1-21.1)	13.6 (8.4-21.6)

Abbreviations: AE-PCOS, Androgen Excess and Polycystic Ovary Syndrome Society; NIH, National Institutes of Health.

### Phenotypes

**Figure 2.**
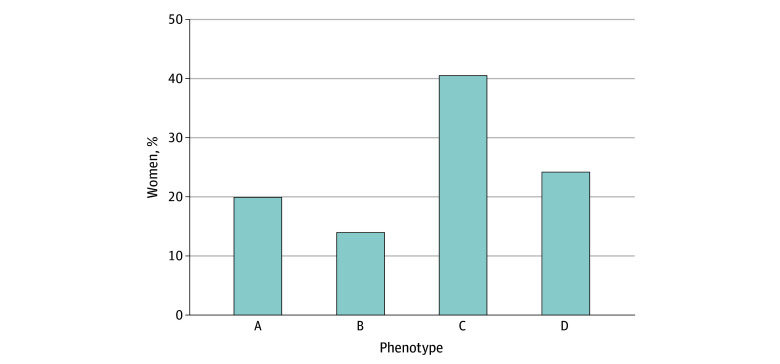
Different Phenotypes of Polycystic Ovary Syndrome Phenotype A includes hyperandrogenism, oligomenorrhea, and polycystic ovarian morphology; phenotype B includes hyperandrogenism and oligomenorrhea; phenotype C includes hyperandrogenism and polycystic ovarian morphology; and phenotype D includes oligomenorrhea and polycystic ovarian morphology.

Overall, PCOS phenotype C was the most prevalent (501 [40.8%]), followed by phenotypes D (301 [24.6%]), A (247 [20.2%]), and B (175 [14.3%]) (Figure 2). In the non-PCOS subgroup, 492 exhibited partial phenotypes, including hyperandrogenism in 257, oligomenorrhea in 75, and PCOM in 160. These individuals did not meet PCOS criteria, and we classified them as having pre-PCOS. The women labeled as having pre-PCOS had characteristics different from both the PCOS group and healthy women as described in Table 1.

### Comorbidities

**Figure 3.**
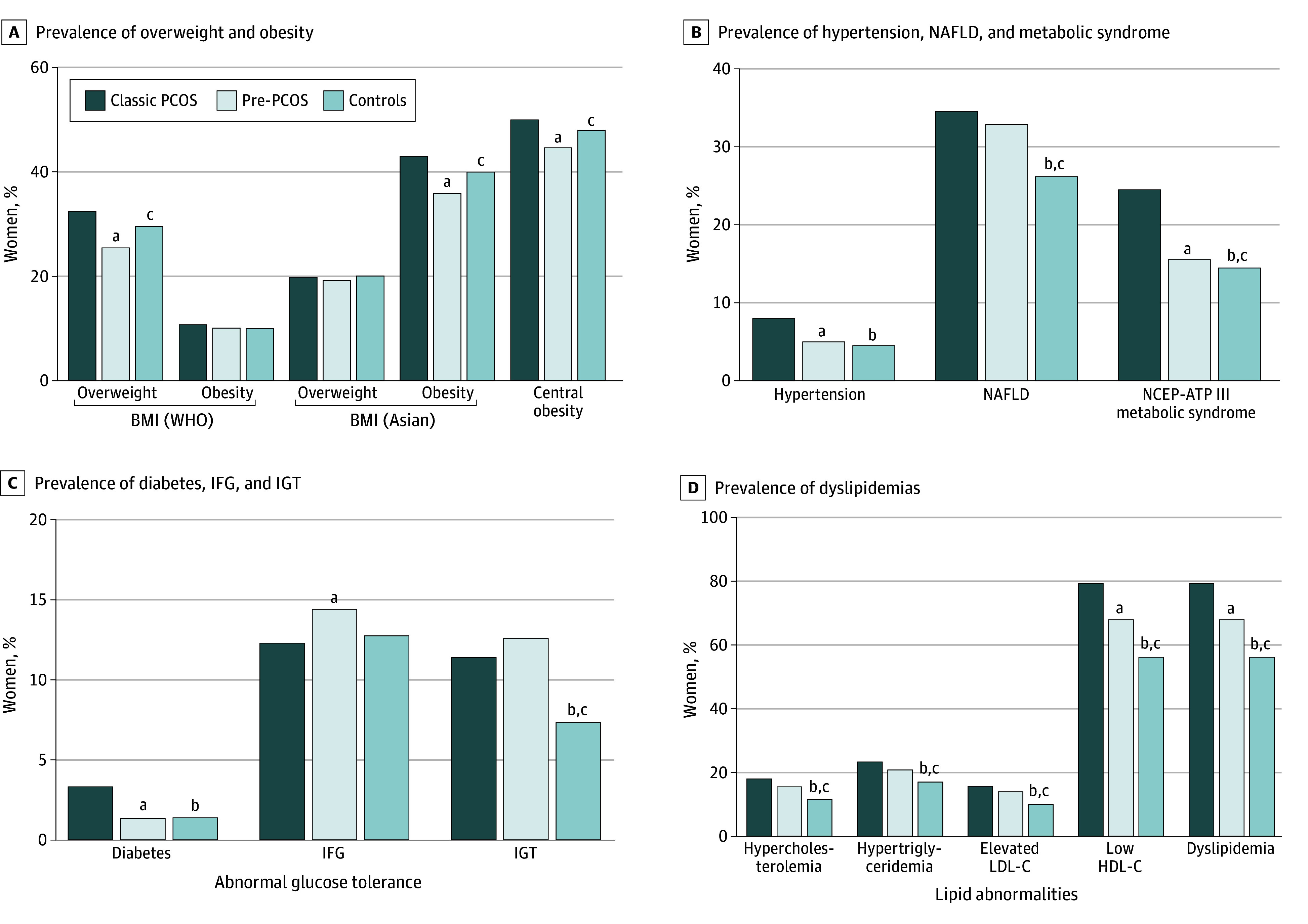
Burden of Comorbidities in Women With Polycystic Ovary Syndrome (PCOS), Women With Pre-PCOS, and Healthy Women BMI indicates body mass index (calculated as weight in kilograms divided by height in meters squared); HDL-C, high-density lipoprotein cholesterol; IFG, impaired fasting glucose; IGT, impaired glucose tolerance; LDL-C, low-density lipoprotein cholesterol; NAFLD, nonalcoholic fatty liver disease; NCEP-ATP III, National Cholesterol Education Program Adult Treatment Panel III; WHO, World Health Organization. ^a^*P* < .05 for classic PCOS vs pre-PCOS. ^b^*P* < .05 for classic PCOS vs healthy. ^c^*P* < .05 for pre-PCOS vs healthy.

Patient comorbidities are shown in Figure 3. Among 1224 women with PCOS, 392 (32.0%) were classified as having overweight and 134 (10.9%) as having obesity according to World Health Organization criteria. However, when Asian cut-offs were applied, 245 (20.0%) had overweight and 529 (43.2%) had obesity. Comparatively, among women with pre-PCOS (n = 492), 95 (19.3%) had overweight and 178 (36.2%) had obesity. Similarly, hypertension was present in 101 (8.3%) and 26 (5.3%) of women with PCOS and pre-PCOS, respectively.

Among women with PCOS, 305 (24.9%) had metabolic syndrome, 403 (32.9%) had NAFLD, and 1126 (91.9%) had any dyslipidemia. Conversely, among women with pre-PCOS, 78 (15.9%) had metabolic syndrome, 163 (33.1%) had NAFLD, and 390 (79.3%) had dyslipidemia.

In the PCOS group, 41 (3.3%) had diabetes, 152 (9.1%) had impaired fasting glucose, and 111 (11.5%) had impaired glucose tolerance. In contrast, in the pre-PCOS group, 7 (1.4%) had diabetes, 71 (14.5%) had impaired fasting glucose, and 62 (12.6%) had impaired glucose tolerance.

## Discussion

Polycystic ovarian syndrome encompasses various reproductive and metabolic issues that may have transgenerational effects.^52^ Prevalence rates of PCOS vary widely both globally and within India. This nationwide study reveals a high prevalence of PCOS across the country. Additionally, most of those affected also experienced comorbidities, such as hypertension, metabolic syndrome, abnormal glucose tolerance, obesity, and dyslipidemia, which contribute to the increasing burden of noncommunicable diseases in India.

On the basis of the NIH 1990 criteria, PCOS prevalence in India was 7.2%, whereas it reached 19.6% with the Rotterdam 2003 criteria. Globally, few large-scale studies have assessed PCOS prevalence, with rates varying based on the criteria used.^22,23,26,29,53^ In the US, among 400 premenopausal women evaluated before employment, the prevalence of PCOS was 6.6% by the Rotterdam 2003 criteria.^24^ In Brazil, 859 women aged 18 to 45 undergoing cervical cancer screening had a PCOS prevalence of 8.5% by the same criteria.^26^ The prevalence reported from Europe was higher at 16.6% (Rotterdam 2003 criteria) among female employees of a general hospital.^54^ Conversely, in Turkey, PCOS prevalence ranged from 6% (NIH 1990 criteria) to 19.9% (Rotterdam 2003 criteria) among women aged 18 to 45 years working at a government institute.^7^ In Asia, PCOS prevalence ranges from 5% to 19%, depending on ethnicity. In the Tehran Lipid and Glucose Study (n = 1960), prevalence rates according to different diagnostic criteria were 13.6% (NIH 1990), 19.4% (Rotterdam 2003), and 17.8% (AE-PCOS), respectively.^29^ In a community-based study in Sri Lanka, 3030 women aged 15 to 39 years were surveyed, and the estimated prevalence of PCOS was 6.3% by Rotterdam 2003 criteria.^23^ In 2013, a study of 15 924 individuals in major Chinese provinces found a PCOS prevalence of 5.6% using Rotterdam 2003 criteria.^22^ Ten years later, the authors noted a prevalence increase to 7.8%.^53^ Despite large sample sizes, these studies from Asia faced issues such as enrolling perimenopausal women, inconsistent hirsutism criteria, limited parameter analysis, and nonuniform serum and ultrasonography cut-offs, resulting in a lower prevalence compared with ours. Unlike previous Indian studies, which were limited to a particular area, had small sample sizes, and used inconsistent methods, resulting in wide-ranging PCOS prevalence rates from 2% to 35%,^30,31,32,33,34,55^ the current study extensively covered almost all major regions of India. A study from Andra Pradesh (South India) reported a prevalence of 9.3% (Rotterdam 2003 criteria) among college girls (n = 460) aged 15 to 18 years.^30^ In Mumbai (Western India), the PCOS prevalence among adolescent girls (n = 778) was 22.5% by Rotterdam 2003 criteria.^32^ Conversely, in a community-based survey (n = 500), the prevalence was reported as 8.2% from Bhopal (Central India).^33^ In Lucknow (North India), the PCOS prevalence was estimated at 3.7% among girls aged 18 to 25 years (n = 1520) using the NIH 1990 criteria.^34^ Similarly, another study estimated prevalence at 4.2% (Rotterdam 2003 criteria) from Haryana (North India) among 2400 reproductive age women.^55^ Both these studies were based on clinical data and lacked complete biochemical and radiological evaluation. In Kashmir (Northern India), the PCOS prevalence was exceptionally high at 35.3% among 3300 women aged 15 to 40 years, possibly due to robust recruitment methods.^36^ Our nationally representative study, which used consistent methods and thorough evaluation, revealed a higher PCOS prevalence, which could be attributed to India’s high burden of noncommunicable diseases, increased disease awareness, and more sensitive diagnostic tools.^56^

Our study, aligning with prior Indian research, notes a slightly higher PCOS prevalence in urban settings, reflecting urbanization trends.^32,55^ Furthermore, the decrease in PCOS prevalence with age observed in the current study and aligning with previous data^54^ could be attributed to declining ovarian function and decreased androgen production by ovaries.^54,57,58^

One contrasting feature of the current study is the predominance of phenotypes C and D in India, compared with phenotype A,^59,60^ stemming from heightened awareness among women, improved accessibility to ultrasonography, better resolution of ultrasonography machines, and enhanced sonographer training, which also has contributed to higher estimates of PCOS prevalence. In addition, transabdominal ultrasonography was used, with the cut-off for follicle count (size, 2-8 mm) greater than 12, which could have overestimated the prevalence of PCOM. Furthermore, a significant number of women in the current study exhibited oligomenorrhea, hyperandrogenism, or PCOM and were diagnosed as having pre-PCOS. These women with pre-PCOS have higher rates of hypertension, metabolic syndrome, NAFLD, and dyslipidemia compared with healthy women. As documented earlier, these women have metabolic irregularities falling between those of women with PCOS and healthy individuals,^2,61^ which emphasizes the concept of partial phenotypes or evolving PCOS, requiring periodic follow-up.

Consistent with prior research, our study highlights a significant prevalence of comorbidities among women with PCOS, including obesity, metabolic syndrome, NAFLD, hypertension, and abnormal glucose tolerance.^2,8,13,62,63^ Approximately half of women diagnosed with PCOS have overweight or obesity, with approximately one-third exhibiting signs of metabolic syndrome and nearly all displaying some form of lipid abnormality. These findings mirror recent research indicating that one-third of Indians have obesity, 80% have dyslipidemia, and a considerable number have diabetes and hypertension.^56^

### Limitations

This study has some limitations, such as (1) inclusion of fewer states and union territories, (2) higher dropout rates due to the COVID-19 pandemic, (3) lack of estimation of androstenedione and sex hormone–binding globulin, (4) use of transabdominal ultrasonography to estimate ovarian volume rather than a transvaginal approach, and (5) use of electrochemiluminescence immunoassay rather than liquid chromatography–mass spectrometry for androgen estimation. It is also worth noting that recall bias in menstrual history reporting cannot be completely ruled out as a potential source of error in our study.

## Conclusions

In this cross-sectional study of reproductive-age women recruited across India, the prevalence of PCOS was high, with phenotype C being predominant. The majority of these women with PCOS had obesity, dyslipidemia, NAFLD, and dysglycemia. Some women had hyperandrogenism, oligomenorrhea, or PCOM only and were considered to have pre-PCOS with significant metabolic aberrations. These findings are crucial for developing preventive and therapeutic strategies, potentially integrating PCOS management into national health programs.

## Notes

Supplement 1. **Nonauthor Collaborators.** PCOS Study Group

Supplement 2.
Data Sharing Statement

## References

[1] Teede HJ, Tay CT, Laven JJE, ; International PCOS Network. Recommendations from the 2023 international evidence-based guideline for the assessment and management of polycystic ovary syndrome. Eur J Endocrinol. 2023;189(2):G43-G64. doi:10.1093/ejendo/lvad096 37580861

[2] Ganie MA, Rashid A, Baba MS, . Pre-polycystic ovary syndrome and polymenorrhoea as new facets of polycystic ovary syndrome (PCOS): evidences from a single centre data set. Clin Endocrinol (Oxf). 2023;99(6):566-578. doi:10.1111/cen.14964 37656656

[3] Brower M, Brennan K, Pall M, Azziz R. The severity of menstrual dysfunction as a predictor of insulin resistance in PCOS. J Clin Endocrinol Metab. 2013;98(12):E1967-E1971. doi:10.1210/jc.2013-2815 24092831 PMC3849664

[4] Balen AH, Conway GS, Kaltsas G, . Polycystic ovary syndrome: the spectrum of the disorder in 1741 patients. Hum Reprod. 1995;10(8):2107-2111. doi:10.1093/oxfordjournals.humrep.a136243 8567849

[5] Apridonidze T, Essah PA, Iuorno MJ, Nestler JE. Prevalence and characteristics of the metabolic syndrome in women with polycystic ovary syndrome. J Clin Endocrinol Metab. 2005;90(4):1929-1935. doi:10.1210/jc.2004-1045 15623819

[6] Zhang HY, Guo CX, Zhu FF, Qu PP, Lin WJ, Xiong J. Clinical characteristics, metabolic features, and phenotype of Chinese women with polycystic ovary syndrome: a large-scale case-control study. Arch Gynecol Obstet. 2013;287(3):525-531. doi:10.1007/s00404-012-2568-z 23108387

[7] Yildiz BO, Bozdag G, Yapici Z, Esinler I, Yarali H. Prevalence, phenotype and cardiometabolic risk of polycystic ovary syndrome under different diagnostic criteria. Hum Reprod. 2012;27(10):3067-3073. doi:10.1093/humrep/des232 22777527

[8] Wijeyaratne CN, Seneviratne R de A, Dahanayake S, . Phenotype and metabolic profile of South Asian women with polycystic ovary syndrome (PCOS): results of a large database from a specialist Endocrine Clinic. Hum Reprod. 2011;26(1):202-213. doi:10.1093/humrep/deq310 21098627

[9] Weerakiet S, Srisombut C, Bunnag P, Sangtong S, Chuangsoongnoen N, Rojanasakul A. Prevalence of type 2 diabetes mellitus and impaired glucose tolerance in Asian women with polycystic ovary syndrome. Int J Gynaecol Obstet. 2001;75(2):177-184. doi:10.1016/S0020-7292(01)00477-5 11684113

[10] Liu X, Zhang J, Wang S. Global, regional, and national burden of infertility attributable to PCOS, 1990-2019. Hum Reprod. 2024;39(1):108-118. doi:10.1093/humrep/dead241 38011904

[11] Kumarendran B, Sumilo D, O’Reilly MW, . Increased risk of obstructive sleep apnoea in women with polycystic ovary syndrome: a population-based cohort study. Eur J Endocrinol. 2019;180(4):265-272. doi:10.1530/EJE-18-0693 30763274 PMC6410684

[12] Kjerulff LE, Sanchez-Ramos L, Duffy D. Pregnancy outcomes in women with polycystic ovary syndrome: a metaanalysis. Am J Obstet Gynecol. 2011;204(6):558.e1-558.e6. doi:10.1016/j.ajog.2011.03.021 21752757

[13] Ganie MA, Dhingra A, Nisar S, . Oral glucose tolerance test significantly impacts the prevalence of abnormal glucose tolerance among Indian women with polycystic ovary syndrome: lessons from a large database of two tertiary care centers on the Indian subcontinent. Fertil Steril. 2016;105(1):194-201.e1, 3. doi:10.1016/j.fertnstert.2015.09.005 26407537

[14] Ganie MA, Khurana ML, Nisar S, . Improved efficacy of low-dose spironolactone and metformin combination than either drug alone in the management of women with polycystic ovary syndrome (PCOS): a six-month, open-label randomized study. J Clin Endocrinol Metab. 2013;98(9):3599-3607. doi:10.1210/jc.2013-1040 23846820

[15] Melin J, Forslund M, Alesi S, . Metformin and combined oral contraceptive pills in the management of polycystic ovary syndrome: a systematic review and meta-analysis. J Clin Endocrinol Metab. 2024;109(2):e817-e836. doi:10.1210/clinem/dgad465 37554096 PMC10795934

[16] Ganie MA, Khurana ML, Eunice M, . Comparison of efficacy of spironolactone with metformin in the management of polycystic ovary syndrome: an open-labeled study. J Clin Endocrinol Metab. 2004;89(6):2756-2762. doi:10.1210/jc.2003-031780 15181054

[17] Zawadzki JK, Dunaif A. Diagnostic criteria for polycystic ovary syndrome: towards a rational approach. In: Dunaif A, Givens JR, Haseltine FP, Merriam GR, eds. Polycystic Ovary Syndrome. Springer; 1992:377-384.

[18] Rotterdam ESHRE/ASRM-Sponsored PCOS Consensus Workshop Group. Revised 2003 consensus on diagnostic criteria and long-term health risks related to polycystic ovary syndrome (PCOS). Hum Reprod. 2004;19(1):41-47. doi:10.1093/humrep/deh098 14688154

[19] Dewailly D, Lujan ME, Carmina E, . Definition and significance of polycystic ovarian morphology: a task force report from the Androgen Excess and Polycystic Ovary Syndrome Society. Hum Reprod Update. 2014;20(3):334-352. doi:10.1093/humupd/dmt061 24345633

[20] Skiba MA, Islam RM, Bell RJ, Davis SR. Understanding variation in prevalence estimates of polycystic ovary syndrome: a systematic review and meta-analysis. Hum Reprod Update. 2018;24(6):694-709. doi:10.1093/humupd/dmy022 30059968

[21] Lo JC, Feigenbaum SL, Yang J, Pressman AR, Selby JV, Go AS. Epidemiology and adverse cardiovascular risk profile of diagnosed polycystic ovary syndrome. J Clin Endocrinol Metab. 2006;91(4):1357-1363. doi:10.1210/jc.2005-2430 16434451

[22] Li R, Zhang Q, Yang D, . Prevalence of polycystic ovary syndrome in women in China: a large community-based study. Hum Reprod. 2013;28(9):2562-2569. doi:10.1093/humrep/det262 23814096

[23] Kumarapeli V, Seneviratne R de A, Wijeyaratne CN, Yapa RM, Dodampahala SH. A simple screening approach for assessing community prevalence and phenotype of polycystic ovary syndrome in a semi-urban population in Sri Lanka. Am J Epidemiol. 2008;168(3):321-328. doi:10.1093/aje/kwn137 18550559

[24] Knochenhauer ES, Key TJ, Kahsar-Miller M, Waggoner W, Boots LR, Azziz R. Prevalence of the polycystic ovary syndrome in unselected black and white women of the southeastern United States: a prospective study. J Clin Endocrinol Metab. 1998;83(9):3078-3082. doi:10.1210/jc.83.9.3078 9745406

[25] Goodarzi MO, Quiñones MJ, Azziz R, Rotter JI, Hsueh WA, Yang H. Polycystic ovary syndrome in Mexican-Americans: prevalence and association with the severity of insulin resistance. Fertil Steril. 2005;84(3):766-769. doi:10.1016/j.fertnstert.2005.03.051 16169421

[26] Gabrielli L, Aquino EMI. Polycystic ovary syndrome in Salvador, Brazil: a prevalence study in primary healthcare. Reprod Biol Endocrinol. 2012;10:96. doi:10.1186/1477-7827-10-96 23173761 PMC3560118

[27] Azziz R, Woods KS, Reyna R, Key TJ, Knochenhauer ES, Yildiz BO. The prevalence and features of the polycystic ovary syndrome in an unselected population. J Clin Endocrinol Metab. 2004;89(6):2745-2749. doi:10.1210/jc.2003-032046 15181052

[28] Asunción M, Calvo RM, San Millán JL, Sancho J, Avila S, Escobar-Morreale HF. A prospective study of the prevalence of the polycystic ovary syndrome in unselected Caucasian women from Spain. J Clin Endocrinol Metab. 2000;85(7):2434-2438. doi:10.1210/jc.85.7.2434 10902790

[29] Farhadi-Azar M, Behboudi-Gandevani S, Rahmati M, . The prevalence of polycystic ovary syndrome, its phenotypes and cardio-metabolic features in a community sample of Iranian population: Tehran Lipid and Glucose Study. Front Endocrinol (Lausanne). 2022;13:825528. doi:10.3389/fendo.2022.825528 35299965 PMC8920974

[30] Nidhi R, Padmalatha V, Nagarathna R, Amritanshu R. Prevalence of polycystic ovarian syndrome in Indian adolescents. J Pediatr Adolesc Gynecol. 2011;24(4):223-227. doi:10.1016/j.jpag.2011.03.002 21600812

[31] Jabeen A, Yamini V, Rahman Amberina A, . Polycystic ovarian syndrome: prevalence, predisposing factors, and awareness among adolescent and young girls of South India. Cureus. 2022;14(8):e27943. doi:10.7759/cureus.27943 36120281 PMC9464521

[32] Joshi B, Mukherjee S, Patil A, Purandare A, Chauhan S, Vaidya R. A cross-sectional study of polycystic ovarian syndrome among adolescent and young girls in Mumbai, India. Indian J Endocrinol Metab. 2014;18(3):317-324. doi:10.4103/2230-8210.131162 24944925 PMC4056129

[33] Gupta M, Singh D, Toppo M, Priya A, Sethia S, Gupta P. A cross sectional study of polycystic ovarian syndrome among young women in Bhopal, Central India. Int J Community Med Public Health. 2018;5(1):95-100. doi:10.18203/2394-6040.ijcmph20175603

[34] Gill H, Tiwari P, Dabadghao P. Prevalence of polycystic ovary syndrome in young women from North India: a community-based study. Indian J Endocrinol Metab. 2012;16(suppl 2):S389-S392. doi:10.4103/2230-8210.104104 23565440 PMC3603088

[35] Deswal R, Narwal V, Dang A, Pundir CS. The prevalence of polycystic ovary syndrome: a brief systematic review. J Hum Reprod Sci. 2020;13(4):261-271. doi:10.4103/jhrs.JHRS_95_18 33627974 PMC7879843

[36] Ganie MA, Rashid A, Sahu D, Nisar S, Wani IA, Khan J. Prevalence of polycystic ovary syndrome (PCOS) among reproductive age women from Kashmir valley: a cross-sectional study. Int J Gynaecol Obstet. 2020;149(2):231-236. doi:10.1002/ijgo.13125 32080845

[37] Rosner W, Auchus RJ, Azziz R, Sluss PM, Raff H. Position statement: utility, limitations, and pitfalls in measuring testosterone: an Endocrine Society position statement. J Clin Endocrinol Metab. 2007;92(2):405-413. doi:10.1210/jc.2006-1864 17090633

[38] Ganie MA, Chowdhury S, Suri V, . Evaluation of the prevalence, regional phenotypic variation, comorbidities, risk factors, and variations in response to different therapeutic modalities among Indian women: proposal for the Indian Council of Medical Research-Polycystic Ovary Syndrome (ICMR-PCOS) Study. JMIR Res Protoc. 2021;10(8):e23437. doi:10.2196/23437 34448720 PMC8433859

[39] World Medical Association. World Medical Association Declaration of Helsinki: ethical principles for medical research involving human subjects. JAMA. 2013;310(20):2191-2194. doi:10.1001/jama.2013.281053 24141714

[40] Yildiz BO, Bolour S, Woods K, Moore A, Azziz R. Visually scoring hirsutism. Hum Reprod Update. 2010;16(1):51-64. doi:10.1093/humupd/dmp024 19567450 PMC2792145

[41] Burke BM, Cunliffe WJ. The assessment of acne vulgaris—the Leeds technique. Br J Dermatol. 1984;111(1):83-92. doi:10.1111/j.1365-2133.1984.tb04020.x 6234917

[42] Ludwig E. Classification of the types of androgenetic alopecia (common baldness) occurring in the female sex. Br J Dermatol. 1977;97(3):247-254. doi:10.1111/j.1365-2133.1977.tb15179.x 921894

[43] Teede HJ, Misso ML, Costello MF, ; International PCOS Network. Recommendations from the international evidence-based guideline for the assessment and management of polycystic ovary syndrome. Hum Reprod. 2018;33(9):1602-1618. doi:10.1093/humrep/dey256 30052961 PMC6112576

[44] WHO Expert Consultation. Appropriate body-mass index for Asian populations and its implications for policy and intervention strategies. Lancet. 2004;363(9403):157-163. doi:10.1016/S0140-6736(03)15268-3 14726171

[45] Expert Panel on Detection, Evaluation, and Treatment of High Blood Cholesterol in Adults. Executive summary of the Third Report of the National Cholesterol Education Program (NCEP) Expert Panel on Detection, Evaluation, and Treatment of High Blood Cholesterol in Adults (Adult Treatment Panel III). JAMA. 2001;285(19):2486-2497. doi:10.1001/jama.285.19.2486 11368702

[46] Grundy SM, Brewer HB Jr, Cleeman JI, Smith SC Jr, Lenfant C; American Heart Association; National Heart, Lung, and Blood Institute. Definition of metabolic syndrome: report of the National Heart, Lung, and Blood Institute/American Heart Association conference on scientific issues related to definition. Circulation. 2004;109(3):433-438. doi:10.1161/01.CIR.0000111245.75752.C6 14744958

[47] American Diabetes Association. Classification and diagnosis of diabetes: standards of medical care in diabetes—2019. Diabetes Care. 2019;42(suppl 1):S13-S28. doi:10.2337/dc19-S002 30559228

[48] Azziz R, Kintziger K, Li R, . Recommendations for epidemiologic and phenotypic research in polycystic ovary syndrome: an androgen excess and PCOS society resource. Hum Reprod. 2019;34(11):2254-2265. doi:10.1093/humrep/dez185 31751476

[49] Kim SI, Yoon JH, Park DC, Yang SH, Kim YI. What is the optimal prolactin cutoff for predicting the presence of a pituitary adenoma in patients with polycystic ovary syndrome? Int J Med Sci. 2023;20(4):463-467. doi:10.7150/ijms.80891 37057215 PMC10087626

[50] Gordon CM, Ackerman KE, Berga SL, . Functional hypothalamic amenorrhea: an Endocrine Society clinical practice guideline. J Clin Endocrinol Metab. 2017;102(5):1413-1439. doi:10.1210/jc.2017-00131 28368518

[51] Bozdag G, Mumusoglu S, Zengin D, Karabulut E, Yildiz BO. The prevalence and phenotypic features of polycystic ovary syndrome: a systematic review and meta-analysis. Hum Reprod. 2016;31(12):2841-2855. doi:10.1093/humrep/dew218 27664216

[52] Risal S, Pei Y, Lu H, . Prenatal androgen exposure and transgenerational susceptibility to polycystic ovary syndrome. Nat Med. 2019;25(12):1894-1904. doi:10.1038/s41591-019-0666-1 31792459

[53] Yang R, Li Q, Zhou Z, . Changes in the prevalence of polycystic ovary syndrome in China over the past decade. Lancet Reg Health West Pac. 2022;25:100494. 35669932 10.1016/j.lanwpc.2022.100494 PMC9162959

[54] Lauritsen MP, Bentzen JG, Pinborg A, . The prevalence of polycystic ovary syndrome in a normal population according to the Rotterdam criteria versus revised criteria including anti-Mullerian hormone. Hum Reprod. 2014;29(4):791-801. doi:10.1093/humrep/det469 24435776

[55] Deswal R, Nanda S, Ghalaut VS, Roy PS, Dang AS. Cross-sectional study of the prevalence of polycystic ovary syndrome in rural and urban populations. Int J Gynaecol Obstet. 2019;146(3):370-379. doi:10.1002/ijgo.12893 31220344

[56] Anjana RM, Unnikrishnan R, Deepa M, ; ICMR-INDIAB Collaborative Study Group. Metabolic non-communicable disease health report of India: the ICMR-INDIAB national cross-sectional study (ICMR-INDIAB-17). Lancet Diabetes Endocrinol. 2023;11(7):474-489. doi:10.1016/S2213-8587(23)00119-5 37301218

[57] de Medeiros SF, Yamamoto MMW, Souto de Medeiros MA, Barbosa BB, Soares JM, Baracat EC. Changes in clinical and biochemical characteristics of polycystic ovary syndrome with advancing age. Endocr Connect. 2020;9(2):74-89. doi:10.1530/EC-19-0496 31905164 PMC6993261

[58] Brown ZA, Louwers YV, Fong SL, . The phenotype of polycystic ovary syndrome ameliorates with aging. Fertil Steril. 2011;96(5):1259-1265. doi:10.1016/j.fertnstert.2011.09.002 21963227

[59] Tripathy P, Sahu A, Sahu M, Nagy A. Metabolic risk assessment of Indian women with polycystic ovarian syndrome in relation to four Rotterdam criteria based phenotypes. Eur J Obstet Gynecol Reprod Biol. 2018;224:60-65. doi:10.1016/j.ejogrb.2018.02.031 29550643

[60] Sachdeva G, Gainder S, Suri V, Sachdeva N, Chopra S. Comparison of the different PCOS phenotypes based on clinical metabolic, and hormonal profile, and their response to clomiphene. Indian J Endocrinol Metab. 2019;23(3):326-331. doi:10.4103/ijem.IJEM_30_19 31641635 PMC6683693

[61] Hassa H, Tanir HM, Yildiz Z. Comparison of clinical and laboratory characteristics of cases with polycystic ovarian syndrome based on Rotterdam’s criteria and women whose only clinical signs are oligo/anovulation or hirsutism. Arch Gynecol Obstet. 2006;274(4):227-232. doi:10.1007/s00404-006-0173-8 16691383

[62] Legro RS, Kunselman AR, Dodson WC, Dunaif A. Prevalence and predictors of risk for type 2 diabetes mellitus and impaired glucose tolerance in polycystic ovary syndrome: a prospective, controlled study in 254 affected women. J Clin Endocrinol Metab. 1999;84(1):165-169. doi:10.1097/00006254-199906000-00019 9920077

[63] Kar S. Anthropometric, clinical, and metabolic comparisons of the four Rotterdam PCOS phenotypes: a prospective study of PCOS women. J Hum Reprod Sci. 2013;6(3):194-200. doi:10.4103/0974-1208.121422 24347934 PMC3853876

