# Supplementary material for: Prevalence, Phenotypes, and Comorbidities of Polycystic Ovary Syndrome Among Indian Women
Source: JAMA Netw Open. 2024 Oct 23;7(10):e2440583. doi: 10.1001/jamanetworkopen.2024.40583 (PMC11581580; doi:10.1001/jamanetworkopen.2024.40583)
Supplement: Supplement 1. — Nonauthor Collaborators. PCOS Study Group [file jamanetwopen-e2440583-s001.pdf]

Supplemental Online Content: Nonauthor Collaborators*First name, last name, and suffix (if applicable) are required and will appear in PubMed.

*Group Name(s): PCOS Study Group

*First Name and Middle Initial(s)	*Last Name	*Suffix (eg, Jr, III)	Academic Degrees	Institution	Location (city, state/province, country)	Role or Contribution, eg, chair, principal investigator	Group (if more than 1 Group listed in the byline) and/or Subgroup (eg, Steering Committee)
Parvaiz	Koul	Dr		SKIMS Srinagar	SRINAGAR	Co-Investigators	
Zafar Amin	Shah	Dr		SKIMS Srinagar	SRINAGAR	Co-Investigators	
Shariq	Masoodi	Dr		SKIMS Srinagar	SRINAGAR	Co-Investigators	
Tabasuum	Parvez	Dr		SKIMS Srinagar	SRINAGAR	Co-Investigators	
Ghulam Nabi	Yatoo	Dr		SKIMS Srinagar	SRINAGAR	Co-Investigators	
Naseer Ahmad	Chow	Dr		SKIMS Srinagar	SRINAGAR	Co-Investigators	
Tariq	Gojwari	Dr		SKIMS Srinagar	SRINAGAR	Co-Investigators	
Muzaffar	Wani	Dr		SKIMS Srinagar	SRINAGAR	Co-Investigators	
Seema	Qayoom	Dr		SKIMS Srinagar	SRINAGAR	Co-Investigators	
Wahid	Khan	Dr		SKIMS Srinagar	SRINAGAR	Co-Investigators	
Rama	Walia	Dr		PGIMER Chandigarh	CHANDIGARH	Co-Investigators	
Deepenkar	De	Dr		PGIMER Chandigarh	CHANDIGARH	Co-Investigators	
Aashima	Arora	Dr		PGIMER Chandigarh	CHANDIGARH	Co-Investigators	
Tulika	Singh	Dr		PGIMER Chandigarh	CHANDIGARH	Co-Investigators	
Neena	Khanna	Dr		AIIMS New Delhi	NEWDELHI	Co-Investigators	
Nitish	Naik	Dr		AIIMS New Delhi	NEWDELHI	Co-Investigators	
Shyam	Prakash	Dr		AIIMS New Delhi	NEWDELHI	Co-Investigators	
Nandita	Gupta	Dr		AIIMS New Delhi	NEWDELHI	Co-Investigators	
Rajesh	Sagar	Dr		AIIMS New Delhi	NEWDELHI	Co-Investigators	
Jai Bhagwan	Sharma	Dr		AIIMS New Delhi	NEWDELHI	Co-Investigators	
Devasenathipathy	Kandasamy	Dr		AIIMS New Delhi	NEWDELHI	Co-Investigators	
Narender Kuber	Bodhey	Dr		AIIMS Raipur	RAIPUR	Co-Investigators	
Eli	Mohapatra	Dr		AIIMS Raipur	RAIPUR	Co-Investigators	
Sabah	Siddiqui	Dr		AIIMS Raipur	RAIPUR	Co-Investigators	
Gourisankar	Kamilya	Dr		IPGMER Kolkata	KOLKATA	Co-Investigators	
Archana	Singh	Dr		IPGMER Kolkata	KOLKATA	Co-Investigators	
Pradip	Mukhopadhyay	Dr		IPGMER Kolkata	KOLKATA	Co-Investigators	
Nehar Ranjan	Sarkar	Dr		IPGMER Kolkata	KOLKATA	Co-Investigators	
Alice Abraham	Ruram	Dr		NEIGRIHMS, Shillong	SHILLONG	Co-Investigators	
Jessy	Abraham	Dr		NEIGRIHMS, Shillong	SHILLONG	Co-Investigators	
Donboklang	Lynser	Dr		NEIGRIHMS, Shillong	SHILLONG	Co-Investigators	
Manika	Agrawal	Dr		NEIGRIHMS, Shillong	SHILLONG	Co-Investigators	
Laksman	Rao	Dr		OMC Hyderabad	HYDERABAD	Co-Investigators	
Sudha	Bindu	Dr		OMC Hyderabad	HYDERABAD	Co-Investigators	

Supplemental Online Content: Nonauthor Collaborators*First name, last name, and suffix (if applicable) are required and will appear in PubMed.

***First Name and Middle Initial(s)**	***Last Name**	***Suffix (eg, Jr, III)**	Academic Degrees	Institution	Location (city, state/province, country)	Role or Contribution, eg, chair, principal investigator	Group (if more than 1 Group listed in the byline) and/or Subgroup (eg, Steering Committee)
Malathi	Ponnuru	Dr		OMC Hyderabad	HYDERABAD	Co-Investigators	
Nadeem	Ahmad	Dr		OMC Hyderabad	HYDERABAD	Co-Investigators	
Sujatha	Rani	Dr		OMC Hyderabad	HYDERABAD	Co-Investigators	
Aleem Ahmad	Khan	Dr		MHRT Hyderabad	HYDERABAD	Co-Investigators	
Sultan Rizwan	Ahmad	Dr		MHRT Hyderabad	HYDERABAD	Co-Investigators	
Prabhakar	Rao	Dr		MHRT Hyderabad	HYDERABAD	Co-Investigators	
Nirmala	C	Dr		MHRT Hyderabad	HYDERABAD	Co-Investigators	
Anuja Elizabeth	George	Dr		GMC Trivandrum	TRIVANDRUM	Co-Investigators	
Jayasree	Leelamma	Dr		GMC Trivandrum	TRIVANDRUM	Co-Investigators	
Jayakumari		Dr		GMC Trivandrum	TRIVANDRUM	Co-Investigators	
Gaurav	Thakur	Dr		ICMR New Delhi	NEW DELHI	Research staff	
Saba	Noor	Dr		ICMR New Delhi	NEW DELHI	Research staff	
Khursheed	Paddar	Dr		SKIMS Srinagar	SRINAGAR	Research staff	
Gaivee Vinam	Meshram	Dr		AIIMS Raipur	RAIPUR	Junior Medical officer	
Shouvik	Choudhary	Dr		IPGMER Kolkata	KOLKATA	Junior Medical officer	
Aafia	Rashid	Dr		SKIMS Srinagar	SRINAGAR	Research staff	
Wasia	Showkat	Dr		SKIMS Srinagar	SRINAGAR	Research staff	
Rohina	Bashir	Dr		SKIMS Srinagar	SRINAGAR	Research staff	
Rabiya	Rashid	Dr		SKIMS Srinagar	SRINAGAR	Research staff	
Jabish	Manzoor			SKIMS Srinagar	SRINAGAR	Research staff	
Nisar	Ahmad			SKIMS Srinagar	SRINAGAR	Research staff	
Pieu	Adhikary			IPGMER Kolkata	KOLKATA	Research staff	
Subhasish	Pramanik			IPGMER Kolkata	KOLKATA	Research staff	
Rahul	Harish	Dr		IPGMER Kolkata	KOLKATA	Junior Medical officer	
Mudasir	Makhdoomi	Dr		SKIMS Srinagar	SRINAGAR	Research staff	
Mudasir	Fayaz			SKIMS Srinagar	SRINAGAR	Research staff	
Nafeez	Rehman			SKIMS Srinagar	SRINAGAR	Research staff	
Neha	Ravi			AIIMS New Delhi	NEWDELHI	Research staff	
Ajay	Kumar			AIIMS New Delhi	NEWDELHI	Research staff	
Siffali	Chandrakar			AIIMS Raipur	RAIPUR	Research staff	
Nithlesh	Kumar			AIIMS Raipur	RAIPUR	Research staff	
Sudipta	Banerjee			AIIMS Raipur	RAIPUR	Research staff	

Supplemental Online Content: Nonauthor Collaborators*First name, last name, and suffix (if applicable) are required and will appear in PubMed.

***First Name and Middle Initial(s)**	***Last Name**	***Suffix (eg, Jr, III)**	Academic Degrees	Institution	Location (city, state/province, country)	Role or Contribution, eg, chair, principal investigator	Group (if more than 1 Group listed in the byline) and/or Subgroup (eg, Steering Committee)
Humaira	Minhaj	Dr		MHRT Hyderabad	HYDERABAD	Research staff	
Balaji	Bhaskar	Dr		MHRT Hyderabad	HYDERABAD	Research staff	
Shaik	Iqbal			MHRT Hyderabad	HYDERABAD	Research staff	
Ram	Babu			MHRT Hyderabad	HYDERABAD	Research staff	
Arya	Suresh			GMC Trivandrum	TRIVANDRUM	Research staff	
RS	Sharma	Dr		ICMR New Delhi	NEWDELHI	supporting team	
Shalini	Singh	Dr		ICMR New Delhi	NEWDELHI	supporting team	
Malabika	Roy	Dr		ICMR New Delhi	NEWDELHI	supporting team	
Reeta	Rasilly	Dr		ICMR New Delhi	NEWDELHI	supporting team	
Nomita	Chandiok	Dr		ICMR New Delhi	NEWDELHI	Junior Medical officer	
Mehnaaz	Rajab	Dr		SKIMS Srinagar	SRINAGAR	Junior Medical officer	
Arifa	Reshi	Dr		SKIMS Srinagar	SRINAGAR	Research staff	
Deeba	Farhat	Dr		SKIMS Srinagar	SRINAGAR	Research staff	
Abid	Rashid			SKIMS Srinagar	SRINAGAR	Research staff	
Nusrat	Jahan			SKIMS Srinagar	SRINAGAR	Research staff	
Shavini	Choudhary			PGIMER Chandigarh	CHANDIGARH	Research staff	
Naila	Mohiudin	Dr		MHRT Hyderabad	HYDERABAD	Research staff	
Samala	Ranjith			OMC Hyderabad	HYDERABAD	Research staff	
Khalid ul Islam	Rather	Dr		SKIMS Srinagar	SRINAGAR	stastistical Assistant	
