# Supplementary material for: Prevalence, Phenotypes, and Comorbidities of Polycystic Ovary Syndrome Among Indian Women
Source: JAMA Netw Open. 2024 Oct 23;7(10):e2440583. doi: 10.1001/jamanetworkopen.2024.40583 (PMC11581580; doi:10.1001/jamanetworkopen.2024.40583)
Supplement: Supplement 2. — Data Sharing Statement [file jamanetwopen-e2440583-s002.pdf]

Data Sharing Statement

Ganie. Prevalence, Phenotypes, and Comorbidities of Polycystic Ovary Syndrome Among Indian Women. *JAMA Netw Open*. Published October 23, 2024.
doi:10.1001/jamanetworkopen.2024.40583

Data
Data available: Yes
Data types: Deidentified participant data
How to access data: ashraf.endo@gmail.com
When available: With publication

Supporting Documents
Document types: None

Additional Information
Who can access the data: To researchers on reasonable request
Types of analyses: for any purpose
Mechanisms of data availability: with signed data access agreement
Any additional restrictions: NONE
